# Commissure leaflet prolapse closely mimics anterior mitral leaflet perforation in 2-D image of transesophageal echocardiography

**DOI:** 10.1186/s40981-023-00659-z

**Published:** 2023-10-16

**Authors:** Kazuto Miyata, Sayaka Shigematsu, Naoki Miyayama

**Affiliations:** Department of Anesthesia, New Heart Watanabe Institute, Hamadayama 3-19-11, Tokyo, Suginami-Ku Japan

**Keywords:** Diverse mitral regurgitation, Regurgitation orifice, Mitral valve perforation

## Abstract

**Background:**

Precise diagnosis of mitral valve regurgitation is challenging, particularly for distinguishing between commissure leaflet prolapse and anterior leaflet perforation, based exclusively on 2-dimensional (2-D) imaging by transesophageal echocardiography.

**Case 1:**

Two mitral regurgitation jets suggesting anterior leaflet perforation, but no regurgitation orifices, were observed in the mid esophageal (ME) 4-chamber view. Multiple 2-D and 3-dimensional (3-D) images revealed prolapse of the anterior (A3) leaflet and posterior commissure, not anterior leaflet perforation.

**Case 2:**

A regurgitation jet suggesting an anterior leaflet prolapse with a regurgitation orifice was observed in ME long-axis view. Multiple 2-D and 3-D images showed only anterior commissure prolapse, but no signs of anterior leaflet perforation.

**Conclusions:**

A regurgitant jet caused by commissure leaflet prolapse closely resembles anterior leaflet perforation in 2-D imaging. Careful evaluation of multiple 2-D and 3-D images, as well as of the regurgitation orifices, is crucially important for making an accurate diagnosis.

## Background

Transesophageal echocardiography (TEE) is commonly used for detecting the site of lesions, mechanisms, and for evaluating the severity to make an accurate diagnosis of mitral regurgitation (MR) during surgery [1, 2]. However, diagnosing complex mitral valve lesions can be challenging [3].

In cases of commissure leaflet prolapse, there may be a suspicion of anterior leaflet perforation based on specific two-dimensional (2-D) images, but further evaluation using multiple 2-D and three-dimensional (3-D) images can rule out anterior leaflet perforation.

To differentiate between commissure leaflet prolapse and anterior leaflet perforation, a thorough evaluation of multiple 2-D and 3-D views is crucial for an accurate diagnosis. Moreover, emphasizing the assessment of regurgitation orifice can further enhance diagnostic accuracy.

## Case presentation

### Case 1

A 50-year-old male scheduled for minimally invasive mitral valve repair due to severe mitral regurgitation. Preoperative evaluation revealed an ejection fraction (EF) of 67% and severe MR classified as Carpentier 2 type with A3, PC, and P3 prolapses.

**Fig. 1:**
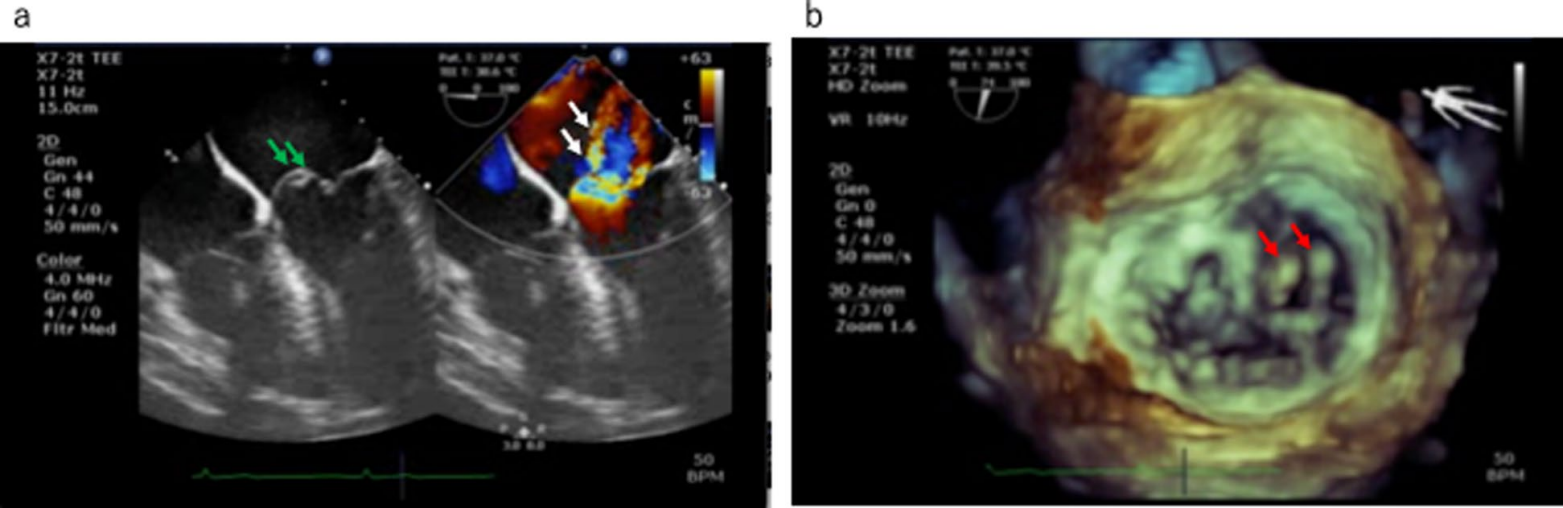
**a** Mid-esophageal four chamber view and **b** three-dimensional transesophageal echocardiography in case 1. **a** Aneurysm-like change (green arrows) in the center of the anterior leaflet (left), with two regurgitation jets apparently in the center of it (white arrows) (right), but with no regurgitation orifices. **b** Three-dimensional view demonstrates prolapse of the anterior (A3) and of posterior commissure segment (red arrows)

During general anesthesia, TEE was performed using a Philips CX50 (Philips Medical Systems, Andover, MA, USA) system. The mid-esophageal (ME) 4-chamber view exhibited aneurysm-like findings in the valve in the middle of the anterior leaflets (Fig. 1a, green arrow). Color Doppler imaging indicated two mitral regurgitant jets: one from the coaptation between the anterior leaflet and posterior leaflet and the second from the central part of the anterior leaflet, suggestive of anterior leaflet perforation (Fig. 1a, white arrow). However, further evaluation of the two regurgitation jets revealed only one regurgitation orifice located between the anterior and posterior leaflets, indicating an A3 prolapse. The 3-D image confirmed A3 and PC prolapse (Fig. 1b, red arrow), and other 2-D views also confirmed A3 and PC prolapse.

After establishing cardiopulmonary bypass (CPB), the aorta was clamped. A3 and PC prolapse without anterior leaflet perforation were elucidated for surgical view. After artificial chordae reconstruction at A3 and PC, a full-ring Memo 3-D 32-mm mitral ring (Sorin Group, Milan, Italy) was used for annuloplasty. TEE performed after CPB weaning showed no residual regurgitation jet.

### Case 2

**Fig. 2:**
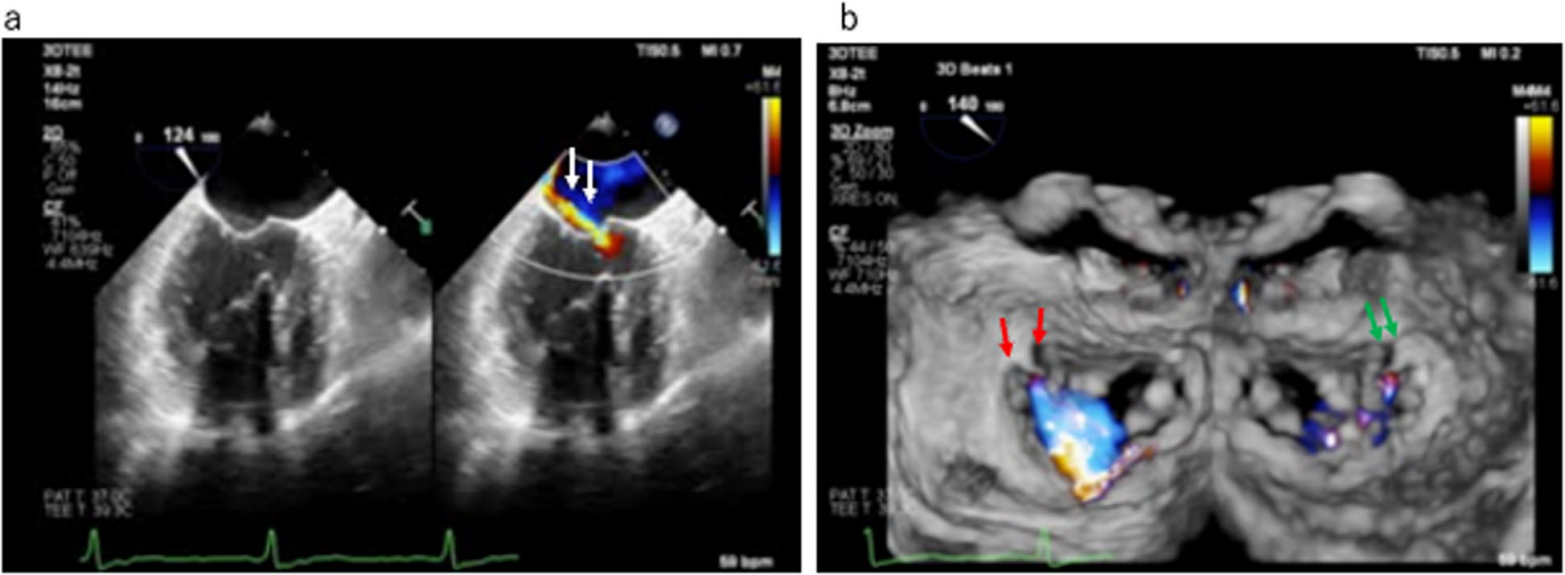
**a** Mid-esophageal long-axis view with a counterclockwise rotation of the probe and **b** 3-D color Doppler image in case 2. **a** Regurgitation jets apparently from an orifice in the center of the anterior leaflet (white arrows). **b** Regurgitant jets (red arrows) and the regurgitation orifice (green arrows) attributed to the prolapse of the anterior commissure (left: enface view, right: enface view from the left ventricular side)

A 65-year-old man scheduled for aortic valve replacement with minimal right thoracotomy due to severe aortic regurgitation (AR) caused by infective endocarditis (IE). Preoperative transthoracic echocardiography indicated an EF of 45% with severe AR and moderate MR. TEE using the Philips Epic 7C (Philips Medical Systems Andover, MA, USA) system was performed after general anesthesia induction. The ME long-axis (LAX) view showed severe AR due to the bending of the right coronary cusp with a hyperechoic mass, indicating a healed IE. Counterclockwise rotation of the ME-LAX probe revealed moderate mitral regurgitation from the center of the anterior leaflet with a regurgitation orifice, raising suspicion of anterior leaflet perforation (Fig. 2a, white arrow). However, the 3-D color image showed only prolapse of the AC, indicating that the regurgitation orifice was due to AC prolapse (Fig. 2b, red arrow, green arrow). After establishing CPB for moderate MR and IE, the mitral valve was directly observed. Intraoperatively, only prolapse of the AC was observed, with no evidence of IE extending to the mitral valve or perforation of the anterior leaflet. Subsequently, edge-to-edge repair of the AC commissure was performed, followed by aortic valve replacement using a Regent 23-mm prosthetic valve (SJM St. Paul, MN, USA). Post-cardiopulmonary weaning, no residual MR findings, or prosthetic valve dysfunction were observed.

## Discussion

Two important points are highlighted in these cases. First, anterior leaflet perforation and commissure leaflet prolapse presented similar images in specific 2-D views. Second, to differentiate between these conditions, confirmation from multiple 2-D and 3-D views, as well as assessment of the regurgitation orifice, is crucial.

**Fig. 3:**
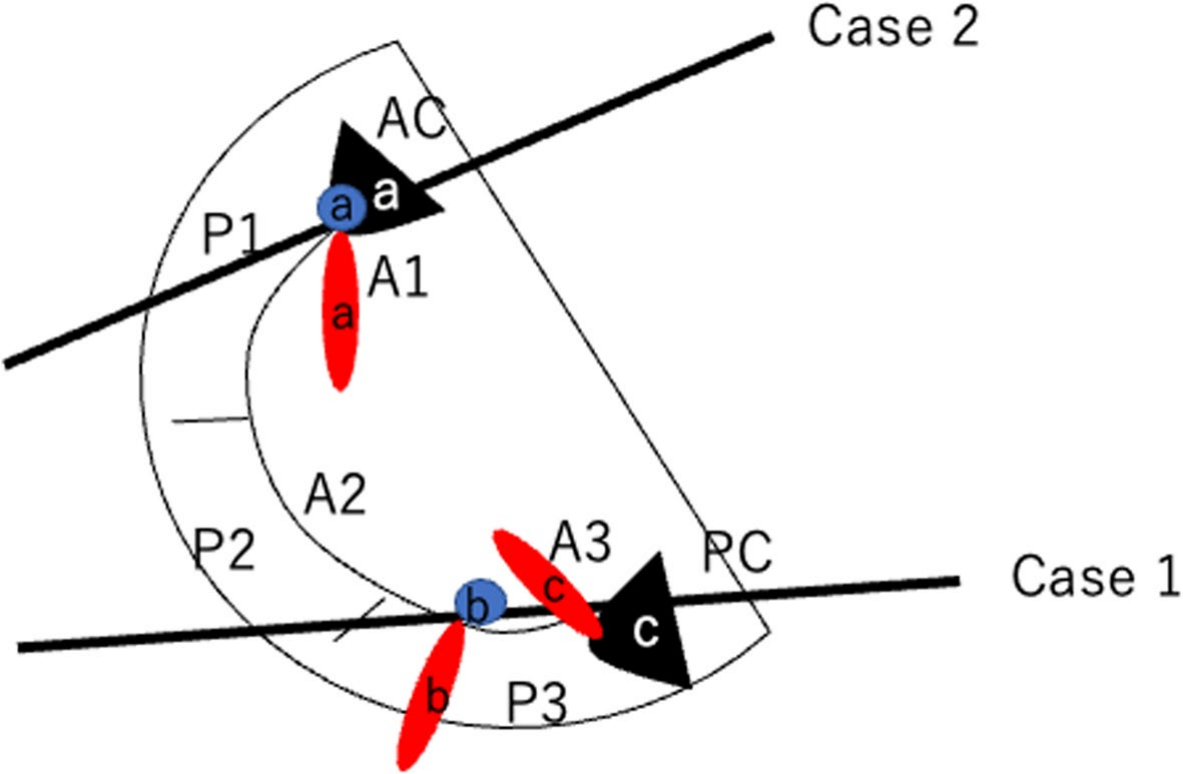
Schematic illustration of the commissure leaflet prolapse (black triangles), regurgitation orifice (blue circles), and mitral regurgitation jet (red ellipses). Case 1: Imaging plane with the probe advanced from the mid-esophageal 4-chamber view. In this image, anterior (A3), posterior (P3), and posterior commissure (PC) (black triangle c) are observed. The redundant PC (black triangle c) was superimposed on the A3. Mitral regurgitation jets due to both A3 (red ellipse b) and PC (red ellipse c) are shown, but the regurgitation orifice (blue circles b) was only shown on A3. Case 2: Imaging plane with the probe with a counterclockwise rotation from the mid-esophageal long-axis view. In this image, A1 and anterior commissure (AC) (black triangle a) are shown. Prolapse of the AC (black triangle a) is superimposed on the anterior leaflet (A1). Mitral regurgitation jet (red ellipse a) due to AC is shown with regurgitation orifice (blue circle a) due to AC prolapse

In the 2-D view, cardiac structures in the same plane are visualized. However, if cardiac structures not on the same plane are redundant or exhibit significant motion, they may appear. Based on this principles, normal commissure leaflets are typically small and not always identified on echocardiography. However, if they become redundant or exhibit substantial motion, such as in cases of prolapse and flail, they may be visualized, even if they are not on same plane (Fig. 3).

In case 1, the prolapse of the redundant PC overlapped with the anterior leaflet, leading to the appearance of A3 and the redundant PC in the same plane. The mitral regurgitation originating from the PC appeared as a perforation of the anterior leaflet (Fig. 1, white arrows). The presence of two regurgitant jets, known as the “crossed sword sign,” suggests the presence of complex lesions [4, 5]. Additionally, In ME-4-chamber view, only one regurgitation orifice was observed on the left ventricular side, originating from the regurgitation jet of A3 (Fig. 3). The regurgitation jet in the middle of the anterior leaflet did not have a regurgitation orifice on the left ventricular side, representing the regurgitation jet caused by PC (Fig. 3).

In case 2, due to the impingement of the aortic regurgitant jet on the mitral anterior leaflet and the presence of IE, expansion of the infection to the mitral valve was suspected. The ME-LAX view with the probe rotated counterclockwise indicated regurgitant jet with regurgitation orifice at the center of the anterior leaflet, involving A1 and P1 segments. In this case, the AC exhibited slight redundancy, and the leaflet motion was intense, suggesting flail motion. Although a regurgitation orifice was observed, no anterior leaflet perforations were noted. The regurgitation jet caused by AC prolapse was visualized as a perforation of the anterior leaflet because, in the counterclockwise rotated image from the LAX, the anterior leaflet (A1) was superimposed on the intense motion of the AC prolapse (Fig. 3). Therefore, in this case, obtaining not only the regurgitation orifice but also multiple 2-D and 3-D images is essential for confirming the diagnosis.

The use of 3-D imaging plays a significant role in the diagnosis of mitral valve morphology and in identifying the prolapse of the main mitral valve. This can be achieved by examining not only the en face view 3-D images but also by rotating the 3-D images from the en face view to different angles, which facilitates the diagnosis of the lesion [1].

However, the volume rate of 3-D TEE color images usually can be low, depending on the equipment model, thereby limiting the diagnostic capabilities of mitral valve lesions solely based on 3-D TEE color images. In case 1, the use of a Philips CX50 system with a 3-D TEE color Doppler image at a very low volume rate did not contribute to the diagnosis of mitral valve regurgitation. In case 2, with a high-volume rate model such as the Philips Epic 7C, the 3-D color Doppler image might not have been able to reveal small regurgitant orifices (Fig. 2).

In conclusion, mitral regurgitation resulting from commissure leaflet prolapse can mimic an anterior leaflet perforation in 2-D images. To ensure accurate diagnosis, it is crucial to rely not only on comprehensive multisectional 2-D and 3-D images but also to emphasize the left ventricular side of the regurgitant orifice. This approach enhances the accuracy of diagnosing mitral valve lesions.

## Abbreviations

TEE: Intraoperative transesophageal echocardiography
MR: Mitral regurgitation
IE: Infective endocarditis
AR: Aortic regurgitation
ME: Mid-esophageal
LAX: Long axis
CPB: Cardiopulmonary bypass
